# Venous Thromboembolism Risk Assessment and Prophylaxis: An Audit-Based Study

**DOI:** 10.7759/cureus.89616

**Published:** 2025-08-08

**Authors:** Asfia N Akhtar, Faizan Hameed, Susan O'Shea

**Affiliations:** 1 Respiratory Medicine, University Hospital Waterford, Waterford, IRL; 2 Internal Medicine, Bon Secours Hospital, Cork, IRL; 3 Haematology, Bon Secours Hospital, Cork, IRL

**Keywords:** anticoagulation, mortality, prescription-accuracy, prevention, venous-thromboprophylaxis

## Abstract

Introduction:Venous thromboembolism (VTE), mainly deep vein thrombosis (DVT) and pulmonary embolism (PE), persists as a critical contributor to hospital-acquired mortality. Despite its largely preventable nature, early 2024 data from Bon Secours Hospital in Cork revealed alarmingly low compliance with VTE prophylaxis protocol.

Aim: This study evaluated the implementation efficacy of VTE risk assessment and prophylaxis in adult hospitalised patients at Bon Secours Hospital, Cork, according to National Institute for Health and Care Excellence (NICE) guidelines.

Methodology: Adult patients (127) at increased risk of VTE, specifically individuals aged ≥60 years, patients undergoing major surgical procedures, and/or patients with comorbidities including active cancer, hypertension, diabetes, immunological disorders, immobility or haematological disorders, across nine wards were included in this study. We also checked for any kind of bleeding risk, like active bleeding or low platelets. Each point was given 1 mark if present, which added to increase risk of VTE. We analysed the data to check the compliance with accurate prescription and completeness of the forms according to the NICE guideline. Exclusion criteria included paediatric, gynaecological, day-case admissions, and discharges within 24 hours of admission. The incomplete/inaccurate risk assessments were reported to improve timely and correct prophylaxis prescription.

Results: Compliance surged with assessment completion, reaching 111 out of 127 (87.39%) with prescription accuracy. Timely assessments within 24 hours accounted for 101 (79.52%), yet 16 (12.64%) remained unassessed. A substantial proportion (103, 80.23%) exhibited one or more VTE risk factors, predominantly advanced age and multiple comorbidities. Anticoagulants, particularly tinzaparin, constituted the principal prophylactic measure, with mechanical prophylaxis employed in only 15 (11.81%). Alarmingly, 22 (17.32%) of high-risk individuals were not prescribed any form of prophylaxis.

Conclusion:This study underscores the transformative impact of enforced protocol adherence and targeted multidisciplinary staff education on VTE prevention. The integration of an electronic health record system with real-time prompts could further ensure prophylaxis within the critical 24-hour window.

## Introduction

Hospital-induced venous thromboembolism (VTE) is a serious medical complication that has been rising in the previous years. About 50-60% of all reported VTEs were hospital-acquired, whether it was a deep vein thrombosis (DVT) or pulmonary embolism (PE) [1-3]. The most common presenting symptoms of the patients who developed VTE include shortness of breath, chest pain, leg swelling with pain and warmth [4].

According to the Health Service Executive statistics by Kirke & Crowley (2018), approximately 9% of Irish people died due to VTEs [5]. However, long-term risk and post-thrombotic syndrome were seen in about 30% of the patients [5]. Venous thromboembolism (VTE) presents a substantial clinical and economic burden, as its treatment-along with the management of long-term sequelae-significantly increases healthcare costs [5]. These cases can be prevented if proper risk assessment is carried out within the first 24 hours of admission and accurate prophylactic intervention for thromboprophylaxis is made. However, pharmacological or mechanical prophylaxis prescription is not just necessary but also needs timely administration [6].

Previous studies have proven that VTE is more common in hospitalised patients than in the community. It was also seen that around 70% of the fatal PEs were reported in non-surgical patients. Patients who have a history of > 3 days of immobility or reduced mobility should be offered prophylaxis. This can only be possible if properly assessed for risk at the time of admission. Every hospital should emphasise VTE risk assessment models, and adequate training should be provided within the hospital to ensure patient safety. However, another important domain is assessing bleeding risk before prescribing. Previous studies suggested that 5.5%-6.4% of those who were admitted with or had ischemic stroke during hospitalisation required holding anti-coagulation until the brain scan was done [7]. In such cases, thromboembolic deterrent (TED) stockings are highly recommended.

Bon Secours Hospital, Cork has its own policy on VTE risk assessment of adult medical and surgical patients, suggesting that every patient admitted acutely or electively should undergo a risk assessment for VTE within 24 hours of admission. It includes a review of the patient’s complete medical and family history, medications, and any risk of bleeding. Guidance for VTE prophylaxis is detailed in the final section of the risk assessment. This policy is similar to that suggested in the National Institute for Health and Care Excellence (NICE) guidelines [2].

This study is designed to thoroughly examine the extent to which VTE risk stratification is actively implemented within the Irish hospital setting. Given that VTE, encompassing both deep vein thrombosis and pulmonary embolism, represents a significant cause of morbidity and mortality in hospitalised patients, accurate identification of individuals at risk is critical to ensuring timely and effective preventative care. This is a cross-sectional hospital-based study which will focus on assessing how healthcare professionals utilise established risk assessment tools to categorise patients according to their likelihood of developing VTE, taking into account a variety of clinical factors such as medical history, mobility status, and comorbid conditions. In parallel, the study aims to evaluate whether prophylactic interventions, ranging from pharmacological approaches like anticoagulants to mechanical methods such as compression devices, are being prescribed and administered appropriately, corresponding to the patient’s assessed risk level. This involves not only verifying adherence to national clinical guidelines but also identifying any deviations or inconsistencies in practice. Furthermore, the study will seek to uncover barriers and facilitators influencing the implementation of risk stratification and prophylaxis protocols, including factors related to healthcare provider knowledge, hospital policies, and resource availability. Through comprehensive data analysis and review of patient management practices, it intends to highlight areas where clinical care may fall short, thereby providing valuable insights for improving VTE prevention strategies. Ultimately, this study aims to contribute to enhancing patient safety outcomes by ensuring that prophylactic measures are effectively targeted and consistently applied to reduce the incidence of VTE-related complications within hospital populations.

## Materials and methods

Study design and setting

This audit was conducted in February 2025 at Bon Secours Hospital, a private, secondary care hospital located in Cork, Ireland.

This is a cross-sectional, retrospective study carried out in a defined period, and the data were compiled with the help of the Quality and Safety Department.

Study population

A sample size of 127 patient records across nine wards was selected. All medical and surgical adult in-patients exceeding 24 24-hour admission window were only eligible for inclusion. These patients were admitted under the care of 32 consultants across 14 specialities. Exclusion criteria included day-case admission, obstetric-gynaecological and paediatric cases, and those discharged within 24 hours of admission. 

Data collection

We used the MEG audit tool, which is provided under an institutional, annually renewed contractual agreement between Bon Secours Health System and MEG Support Tools Ltd. (Dublin, Ireland), with no individual license number or activation key. The use of this platform facilitated standardised data entry and supported consistency in data recording.

Each patient's medication chart was manually reviewed, and relevant data were systematically entered into the digital audit tool using secure hospital-provided login credentials to maintain data privacy and integrity. Each chart had a risk assessment form which included details like age ≥60 years, patients undergoing major surgical procedures, and/or patients with comorbidities including active cancer, hypertension, diabetes, autoimmune condition, immobility or haematological disorders, already on established anticoagulation or any sort of bleeding risks like low platelet or active bleeding were ticked and given one point to each (see Appendices). This form was just before the VTE prophylaxis prescription, and the criteria aligned with the guidelines established by NICE [2], ensuring that all necessary clinical details, such as patient history, comorbidities, and anticoagulation status, are accounted for during the assessment process. We thoroughly checked the bleeding risk assessment as well, which was on the same page.

We then matched it with the prophylaxis prescription page to verify the accuracy and completeness according to the patient's weight and history given. Furthermore, the use of mechanical prophylactic measures, such as TED stockings, was specifically assessed to ensure adherence to recommended preventive practices.

Data analysis

The data collected through the MEG audit tool was exported into a secure database. Descriptive statistics were used to summarise the risk assessment and prescription compliance rate. Categorically, we analysed each variable to rule out the presence or absence of any kind of data that helped us to analyse whether the whole sample size had complete or incomplete risk assessment within the 24-hour admission window. Any incorrect data was highlighted to be included in the result as a loophole in the hospital system. The completeness of prescription was also measured in percentages and summarised depending on distribution. Inter-speciality and inter-ward comparisons can be seen in the Results section with a graphical representation. All those patients who had incorrect/incomplete risk assessment or incorrect/missing prophylaxis prescription were labelled as high risk and notified. 

Ethical clearance was not required as this audit was carried out for a quality improvement initiative. Throughout the study, patients’ confidentiality was maintained. Given that the audit involved a retrospective review of anonymised patient data for the purpose of quality improvement, individual informed consent was deemed unnecessary.

## Results

Demographics details

A total of 127 patients’ records were included in this audit. Demographic data, such as exact age and sex, was not recorded. However, patients were categorised based on age group, specifically whether they were above or below 60 years of age. Out of 127 patients’ records, 90 (70.86%) were > 60 years of age. A limitation of this audit is the absence of detailed demographic data, such as patient sex and exact age. Patients were only categorised based on whether they were over or under 60 years of age, in accordance with VTE risk stratification criteria. While this approach aligned with audit objectives, it limits and prevents subgroup analysis based on sex or age.

Specialities

**Figure 1:**
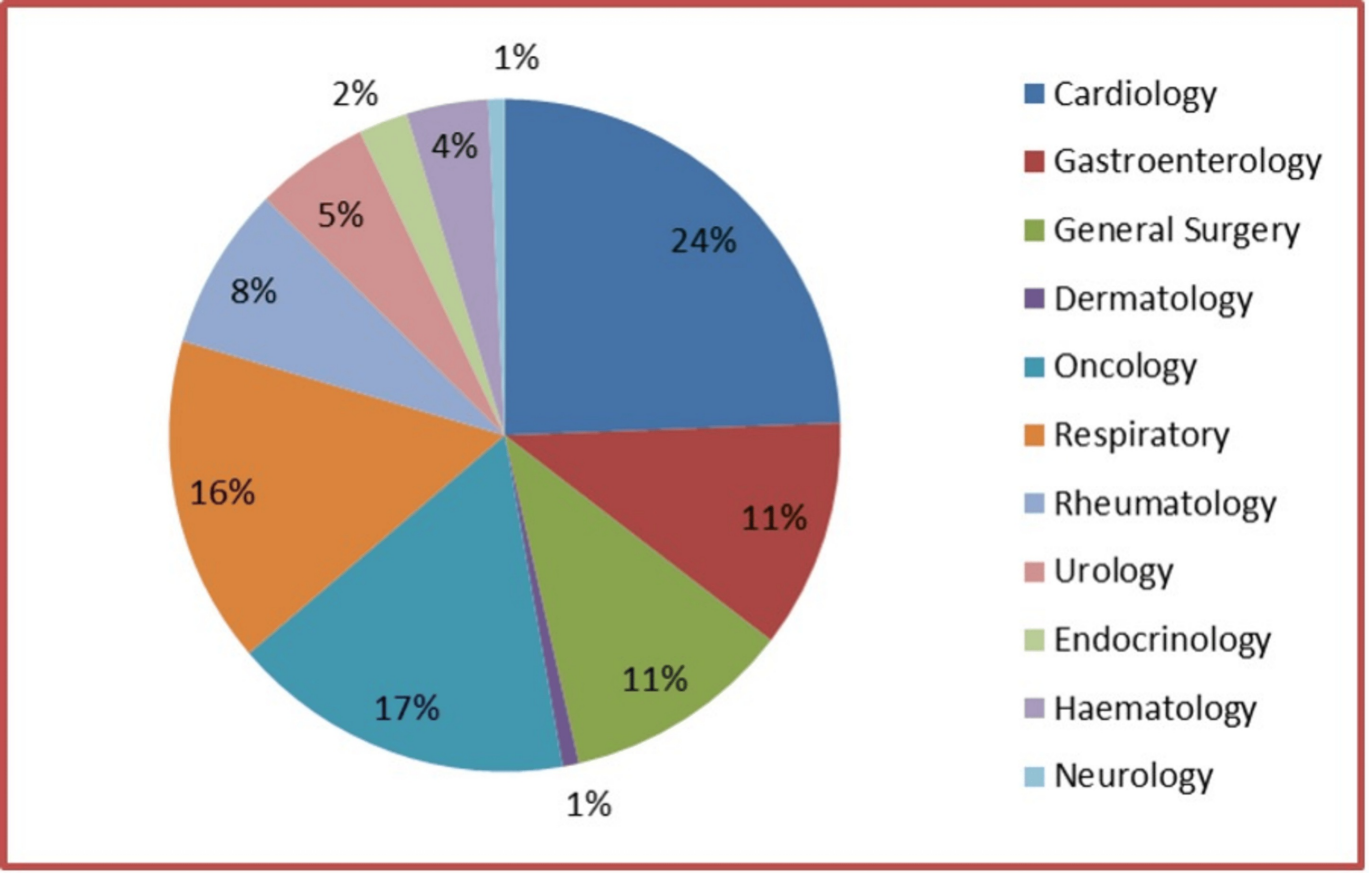
Analysis by percentage of sample size inter-speciality

A sample of 127 charts across nine wards was audited (Figure 1). Overall, 83.46% (n=106) were medical patients and 26.67% (n=21) were surgical patients. The figure below illustrates the distribution of patients audited across various clinical specialities. The largest proportion of patients were from Cardiology, accounting for 24% of the total (mostly already on established anticoagulants for a purpose other than VTE, like atrial fibrillation and mechanical valves). This was followed by Oncology (17%) and Respiratory (16%), which also represented significant portions of the sample. Gastroenterology and General Surgery each contributed 11% of patients, while Rheumatology accounted for 8%. Smaller proportions were observed in Urology (5%), Endocrinology (2%), Dermatology and Neurology (1% each). Haematology and Neurology had the smallest representation at 1% each.

Risk assessment: risk of thrombosis

VTE risk assessment was successfully completed in 87.39% of the patient cohort. Among these, 79.52% (n=101) were assessed within 24 hours of admission, 7.87% (n=10) were evaluated beyond the 24-hour admission window, and the remaining 12.59% (n=16) had no assessment done.

Identified VTE risk factors present at the time of admission are summarised in Table 1. From the analysis, we figured that out of all patients, 10.23% (n=13) had at least one risk factor associated with VTE, while 70% (n=90) had multiple.

**Table 1:** Thrombosis risk factors VTE: venous thromboembolism; HRT: hormone replacement therapy

Thrombosis risk factor	Medical patients (n, %)	Surgical patients (n,%)	Overall (n,%)
Age > 60 years	80 (62.99%)	10 (7.8%)	90 (70.86%)
One or more significant comorbidities (Hypertension, heart failure, diabetes or autoimmune causes)	81 (63.77%)	12 (9.44%)	93 (73.22%)
Active cancer or on cancer treatment	21 (16.53%)	1 (0.79%)	22 (17.32%)
Surgery involving lower pelvis or lower limb with anaesthesia and surgery time >60 minutes	0	2 (1.57%)	2 (1.57%)
Obesity (BMI 30 kg/m^2^)	5 (3.94%)	1 (0.79%)	6 (4.72%)
Total anaesthesia and surgery time >90 minutes	0	2 (1.57%)	2 (1.57%)
History of VTE	2 (1.57%)	1 (0.79%)	3 (2.36%)
Critical care admission	6 (4.72%)	1 (0.79%)	7 (5.51%)
Use of HRT	1 (0.79%)	0 (0%)	1 (0.79%)

The audit identified several key thrombosis risk factors among both medical and surgical patients. The most prevalent risk factor was age greater than 60 years, observed in 62.99% of medical patients and 7.8% of surgical patients, comprising a total of 70.86% across the full cohort. Similarly, the presence of one or more significant comorbidities was highly common, found in 63.77% of medical patients and 9.44% of surgical patients, amounting to 73.22% overall. Active cancer or ongoing cancer treatment was reported in 16.53% of medical patients and 0.79% of surgical patients. Risk factors more specific to the surgical population included procedures involving the lower pelvis or lower limb with anaesthesia and operative time exceeding 60 minutes (1.57%) and surgeries with total anaesthesia and surgical duration exceeding 90 minutes (1.57%). Obesity (defined as a BMI ≥30 kg/m²) was identified in 4.72% of patients, while a history of venous thromboembolism (VTE) was documented in 2.36%. Admission to critical care was noted in 5.51% of patients, predominantly from the medical group. Hormone replacement therapy (HRT) use was observed in a single patient (0.79%). These findings highlight the predominance of age and comorbidities as leading VTE risk factors, particularly within the medical cohort.

Risk assessment: risk of bleeding

The audit also assessed bleeding risk factors among the patient cohort. The most common bleeding risk factor was the use of established therapeutic anticoagulation, identified in 25.98% of medical patients. Notably, none of the surgical patients were on therapeutic anticoagulants. Active bleeding at the time of assessment was present in 3.15% of medical patients, with no such cases observed in the surgical group. Additionally, thrombocytopenia (defined as a platelet count less than 50 × 10⁹/L) was recorded in a single medical patient, accounting for 0.79% of the total population. Overall, all identified bleeding risk factors were found exclusively in medical patients, highlighting the importance of individualised prophylaxis decisions in this subgroup (Table 2).

**Table 2:** Bleeding risk factors

Bleeding risk factor	Medical patients (n, %)	Surgical patients (n,%)	Overall (n,%)
On established therapeutic anticoagulant	33 (25.98%)	0	33 (25.98%)
Active bleeding	4 (3.15%)	0	4 (3.15%)
Platelets <50 x 10^9^/L	1 (0.79%)	0	1 (0.79%)

VTE prophylaxis risk categorisation

Based on the above results, the sample size was categorised according to their combined thrombosis/bleeding risk as follows.

Out of the total patients audited, 5.51% (n=7) were identified as having a minimal risk of venous thromboembolism (VTE), while a significant majority, 81.10% (n=103), were classified as high risk. Additionally, 29.92% (n=38) of patients were found to have a high risk of bleeding, which has important implications for prophylaxis decisions. Notably, 13.38% (n=17) of patients could not be categorised due to incomplete or missing documentation.

Among patients identified as at risk for VTE (n = 103), anticoagulant therapy was the most frequently used form of prophylaxis. Mechanical prophylaxis, specifically thromboembolic deterrent (TED) stockings, was employed in 7.08% (n=9) of cases in conjunction with pharmacological measures, and 4.72% (n=6) of patients as a standalone intervention. 17% (n=22) did not receive any type of VTE prophylaxis and were labelled as at high risk. Two of these patients also had a significant risk of bleeding. Further details regarding the types of prophylaxis administered are provided in Table 3. 

**Table 3:** Prophylaxis types used in at-risk population

Type of prophylaxis	Medical patients	Surgical patients	Overall (n, %)
Total	86 (67.71%)	18 (14.17%)	104 (81.88%)
Pharmaceutical prophylaxis	84 (66.14%)	14 (11.02%)	98 (77.165%)
-Apixaban	20 (15.75%)	0	20 (15.75%)
-Dabigatran	3 (2.36%)	0	3 (2.36%)
-Rivaroxaban	5 (3.93%)	0	4 (3.15%)
-Tinzaparin	56 (44.09%)	14 (11.02%)	70 (55.11%)
Mechanical and pharmaceutical prophylaxis	2 (1.57%%)	7 (5.51%)	9 (7.08%)
Mechanical prophylaxis	2 (1.57%)	4 (3.15%)	6 (4.72%)
Already receiving anticoagulants at therapeutic dose	33 (25.98%)	0	33 (25.98%)
No prophylaxis received	19 (14.96%)	3	22 (17.32%)

Clinical appropriateness of VTE pharmaceutical prophylaxis

A total of 98 patients who received pharmacological VTE prophylaxis were subsequently evaluated for dosing appropriateness based on body weight and estimated glomerular filtration rate (eGFR). All prescriptions were found to be accurate, yielding a 100% compliance rate with dosing guidelines.

Summary of findings

The summary of the above findings suggests that 87.39%% of patients were risk assessed for VTE, 79.52% within 24 hours of admission. While 7.87% were risk assessed > 24 hours of admission. Similarly, 10.23% (n=13) had at least one risk factor associated with VTE, while 70% (n=90) had multiple, with age > 60 years being the topmost element. Others included significant chronic co-morbidities and active or treated cancer history. For surgical patients, the most frequent risk factors were age > 60 years, significant co-morbidities and pelvic or lower limb surgery.

Furthermore, 25.98% of patients were already on established anticoagulants. 81.8% of patients received anticoagulants, of which 100% were prescribed appropriately. Mechanical prophylaxis was used solely in 4.72% of patients and in conjunction with anticoagulants in 7.08% of cases. Of the patients, 17.32% were labelled at high risk of VTE as they were not prescribed any prophylaxis.

## Discussion

The results highlighted significant improvement in the compliance and accuracy of VTE prophylaxis prescription in a hospital setting. It was clearly understood that more than half of the patients were at risk of developing VTE. Prior to intervention and strategic changes in protocols, only 30% of the patients received correct VTE prophylaxis prescription within the first 24 hours of admission. Using NICE guidelines and Bon Secours Hospital Policy, the compliance rate boosted to 81.8% with an accuracy of 100%. This is higher than the rates of compliance ranging from 13% to 64% reported in published studies [8]. This suggests that implementation of proper protocols, mandatory assessment immediately following admission and standardised risk stratification can help in adherence to VTE prophylaxis guidelines as per NICE.

From our audit, we identified that the majority of the patients belonged to the advanced age group, having significant comorbidities. But, one of the previous studies suggested that other variables like acute illness and prior VTE history emerged as stronger predictors [9].

The core issue discussed throughout the audit was risk assessment compliance, but we should not forget that timely administration is also a strong element to prevent VTE in admitted patients. Despite improvements, there were notable gaps in compliance. There were 17.32% of patients not receiving any risk assessment within the first 24-hour critical window. Moreover, 27.57% of patients did not have their bleeding risk assessed, leaving areas that can be further improved in clinical practice. One of the data points from the literature suggests that documentation and decision-making require strengthening and possible digitisation or mandatory EHR prompts [10]. Structured electronic tools and clinical education can bring a dramatic change in the VTE prophylaxis adherence [11]. With the advancement of technology and the development of electronic health records (EHR), it can be an ideal way of reducing the chances of missed VTE prophylaxis prescriptions, as it can prompt the user to complete it without any delay [12]. It is the easiest way of flagging patients who are not risk assessed within a 24-hour window and can also help in improving accuracy. The gaps in assessment, as identified in this audit, could benefit from quality improvement initiatives done quarterly. It is important to monitor continuously and have regular audits on VTE risk assessment to identify the gaps and changes in trend. A robust audit system could help pinpoint areas of improvement and ensure that VTE prevention measures are consistently applied across all patient groups.

It is recommended that all healthcare staff, particularly junior doctors, nurses, and pharmacists, should have continued training on the importance of VTE risk assessment so that in case any of them misses the prescription, the other can highlight and rectification can be done [13]. Regular workshops or teaching sessions should be introduced in the hospital training programme to reinforce best practices.

Moreover, the predominant prophylaxis was a pharmacological one in the at-risk population, with 88% of patients receiving this form. Mechanical prophylaxis was used in a smaller subset of patients. Literature suggests that intermittent pneumatic compressions are superior to graduated compression stockings when used as a single prophylactic agent. It was also recommended that using both in combination can be more efficient in high-risk patients and especially those who are admitted for surgical procedures and cannot receive pharmacological prophylaxis [14].

This study revealed that bleeding risk was not assessed in a significant proportion of patients (26%). It is very important that bleeding risk assessment are carried out on a regular basis as it can contribute to increased morbidity if incorrect prescription is made. This will help to lower complications related to anticoagulant therapy, particularly in contraindicated patients. Previous studies suggested that unnecessary prescriptions can increase the risk of bleeding. Patients on low risk without any clear indication should be avoided for heparin-based prophylaxis; rather, TEDs should be used. It was also recommended that proper guideline-based assessments, like the Padua score, should be used, limiting anticoagulant use to appropriate patients [14]. This will improve patient safety and reduce unnecessary treatment costs.

Patient awareness is an important domain that can also help to improve outcomes. Giving out handouts, education materials and having discussions on VTE prophylaxis could result in greater patient engagement [15], particularly in the advanced aged group and those undergoing surgery involving the lower limb or prolonged anaesthesia.

The primary limitation of this audit is that it addressed VTE risk assessment and prophylaxis accuracy in patients within a 24-hour admission window. It did not measure ongoing adherence throughout the entire hospital stay, nor did it explore the factors causing delay, modification or discontinuation. A notable strength of the audit, however, was the application of a standardised data collection tool aligned with NICE guidelines for the prevention of venous thromboembolism, ensuring methodological consistency and clinical relevance.

## Conclusions

Venous thromboembolism is a very serious complication and requires the attention of every medical practitioner. It is not only a burden to the hospital but can also worsen the quality of life affected by it. We identified from our findings that although significant improvement has been noted, there is still a lot more that can be done. Timely risk assessment and prophylaxis intervention is not just a responsibility of a junior doctor but also senior physicians, nursing staff and every other individual involved in patient care. From our study we can conclude that 87.39%% of patients were risk assessed for VTE, 79.52% within 24 hours of admission. While 7.87% were risk assessed > 24 hours of admission. Similarly, 10.23% had at least one risk factor associated with VTE while 70% had multiple with age > 60 years being the topmost. Furthermore, 25.98% of patients were already on established anticoagulants. 81.8% of patients received anticoagulants of which 100% were prescribed appropriately. Mechanical prophylaxis were used solely in 4.72% of patients and in conjunction with anticoagulants in 7.08% of cases. 17.32% of patients were labelled at high risk of VTE as they were not prescribed any prophylaxis. Patient awareness is an important domain that can also help to improve outcome. Giving out handouts, education materials and having discussion on VTE prophylaxis could result in greater patient engagement, particularly in advanced aged group and those undergoing surgery involving lower limb or prolonged anaesthesia.

## Appendices

**Table 4:** MEG audit tool form MRN: Medical Record Number; VTE: venous thromboembolism; TEDS: thromboembolic deterrent stockings

MRN:		Consultant:									Ward:				
Age		Surgical													Y/N
Gender (M/F)		Medical with reduced mobility (assess for thrombosis & bleeding risk below)													Y/N
Weight		Medical no reduction in mobility (Risk assessment complete no further action required)													Y/N
Thrombosis Risk Assessment															
Was the patient assessed for VTE risk within 24 hours of admission?														Y/N	
Patient Risk Factor					Tick	Admission Risk Factor								Tick	
Age >60 years						Hip or knee replacement									
Active cancer or cancer treatment						Hip fracture									
Dehydration						Total anaesthesia & surgery time >90 minutes									
Known thrombophilia						Surgery involving lower pelvis or lower limb with anaesthesia & surgery time >60 minutes									
Obesity (BMI 30 KG/m2)						Acute surgical admission with inflammatory or intra-abdominal condition									
One or more significant comorbidities						Critical care admission									
Personal history or first-degree relative with a history of VTE						Surgery causing significant reduction in mobility									
Use of hormone replacement therapy															
Use of oestrogen-containing contraceptive therapy															
Varicose veins with phlebitis															
Pregnancy or < 6 weeks post-partum															
Bleeding Risk Assessment															
Patient Risk Factors					Tick	Admission Risk Factors								Tick	
Active bleeding						Neurosurgery, spinal surgery or eye surgery									
Acquired bleeding disorder (e.g., acute liver failure)						Other procedures with high bleeding risk									
Concurrent use of anticoagulant/antiplatelet agents known to increase the risk of bleeding.						Lumbar puncture/epidural /spinal anaesthesia expected within next 12 hrs									
Acute stroke (within 24 hrs)						Lumbar puncture/epidural /spinal anaesthesia within previous 4 hrs									
Platelets <75 x 109/L						Traumatic head injury									
Uncontrolled hypertension (>230 systolic or 120 diastolic or greater)															
Untreated inherited bleeding disorder (e.g. haemophilia, von Willebrand disease)															
Risk Stratification			High Risk of VTE with Low Risk of Bleeding					High Risk of VTE with Significant Risk of Bleeding				Low Risk of VTE			
Was the guidance on the risk stratification followed?			Y/N/N/A				Y/N/N/A					Y/N/N/A			
Was mechanical prophylaxis used?			Y/N	TEDS						Compression devices					
Did the patient receive pharmacological VTE prophylaxis			Y/N	Drug name					Dose				Appropriate? Y/N		
